# Gender differences in physical morbidity in opioid agonist treatment patients: population-based cohort studies from the Czech Republic and Norway

**DOI:** 10.1186/s13011-023-00557-8

**Published:** 2023-07-28

**Authors:** Gabriela Rolová, Desiree Eide, Roman Gabrhelík, Ingvild Odsbu, Thomas Clausen, Svetlana Skurtveit

**Affiliations:** 1 First Faculty of Medicine, Department of Addictology, Charles University, Prague, Czech Republic; 2 Department of Addictology, General University Hospital, Prague, Czech Republic; 3 University of Oslo, Norwegian Centre for Addiction Research, Oslo, Norway; 4 Norwegian Institute of Public Health, Oslo, Norway

**Keywords:** Opioid use disorder, Physical disease, Somatic disease, Record-linkage study, Health registers, Substance use, ICD-10

## Abstract

**Background:**

Physical diseases represent a significant burden for opioid agonist treatment (OAT) patients. This study described physical morbidity in two national cohorts of OAT patients focusing on gender differences.

**Methods:**

This population-based cohort study linking multiple health registers investigated physical diseases (ICD-10) in patients receiving OAT in the Czech Republic (N = 4,280) and Norway (N = 11,389) during 2010–2019. Gender-stratified analysis was performed.

**Results:**

Overall, we found a large burden of physical morbidity across gender groups in OAT patients. In the Czech Republic and Norway, women in OAT had a significantly higher prevalence of physical diseases across most diagnostic chapters, notably genitourinary diseases and neoplasms. Injuries/external causes and infectious/parasitic diseases were among the most common diseases in both women and men. Viral hepatitis accounted for over half of infectious morbidity in women and men in both cohorts.

**Conclusions:**

Our findings support the need for early screening, detection, and treatment of diseases and conditions across organ systems and the integration of health promotion activities to reduce physical morbidity in OAT patients. The gender differences underline the need for a tailored approach to address specific medical conditions.

**Supplementary Information:**

The online version contains supplementary material available at 10.1186/s13011-023-00557-8.

## Introduction

Opioid use disorder (OUD) is a chronic relapsing medical condition associated with increased morbidity and mortality rates. Opioid maintenance treatment (OAT) with methadone, buprenorphine, or buprenorphine/naloxone is a clinically established and effective intervention in reducing patient illicit drug use and improving health and social outcomes [1–4]. However, despite the positive health effects associated with OAT treatment, co-morbid physical diseases still represent a significant burden for OAT patients leading to premature deaths [5–9].

Opioids, both illicit and prescription opioids, play a major role in numerous physiological and pathological processes through their activity on opioid receptors [10]. Their prolonged use may be associated with a range of adverse effects on several organ systems, including central nervous, gastrointestinal, respiratory, immune, digestive, and cardiovascular systems [11]. It has been reported that about half of patients treated with prescription opioids for chronic non-malignant pain experience at least one adverse effect [12]. Opioid-induced adverse effects are well-known, the most severe with potentially fatal consequences being respiratory depression due to sedation [13]. Other common adverse effects associated with opioid use include sedation, dry mouth, nausea, dizziness, vomiting, constipation, impaired sexual functioning, and physical dependence [11, 12, 14].

In general, OAT is considered clinically effective, well-tolerable, and safe treatment for OUD regardless of the potential risk for overdose and widely prevalent adverse effects [4, 15, 16]. In spite of evidence suggesting improved health outcomes in patients after entering OAT, previous studies have shown that the burden of co-morbid physical diseases are still considerably high in this population. Skeie et al. [7, 8] found a significant reduction in healthcare utilization and acute and sub-acute drug-related physical incidents in patients during OAT compared to pre- and post-OAT periods. However, the incidence of non-drug-related incidents and injuries remained unchanged. OAT patients have been also shown to have higher all-cause and cause-specific hospital admission rates than non-dependent individuals [6].

Chronic physical diseases are common among OAT patients and increase with age [9, 17]. In a Norwegian study, 73.5% of long-term OAT patients reported having at least one chronic physical condition, with hepatitis C, asthma, high blood pressure, heart disease, chronic obstructive pulmonary disease (COPD), and diabetes being predominant. Moreover, more than half the patients described having a range of health problems, including impaired memory, headaches, indigestion, dizziness, teeth or gum problems, constipation, and joint pains [9].

The evidence suggests gender differences for health outcomes relating to illicit opioid use. It has been shown that women with OUD are more likely to experience negative health consequences than their male counterparts [18–21]. This higher risk of developing health problems is reflected in both higher morbidity rates and more severe disease manifestation in women [19, 22, 23]. Studies investigating health differences in women and men receiving OAT found that women exhibit poorer physical health, more chronic diseases, and higher hospitalization rates and emergency department (ED) visits than men [6, 9, 17, 24]. However, these studies have been mostly cross-sectional using self-report data or did not evaluate type-specific morbidity.

Therefore, using nationwide health registers, this study aimed to describe and compare gender-specific physical morbidity classified according to the type of disease in two national cohorts of patients receiving OAT. Increasing knowledge of gender-specific disease burdens can help identify the unique health needs of women and men in OAT, leading to the development of tailored interventions to improve their health outcomes.

## Methods

### Study design

This prospective population-based record-linkage cohort study used data from multiple national health registers from the Czech Republic and Norway. The study protocol for the overall registry linkage study can be found in Gabrhelik, Handal [25].

### Setting

This study was carried out in the Czech Republic and Norway. The countries are quite similar in their population characteristics, healthcare systems, and OAT programs.

The Czech Republic and Norway have publicly funded healthcare systems that provide universal coverage to all citizens and legal residents. Healthcare services are largely financed through taxation and employer/employee contributions, and both countries have a decentralized healthcare system with a mix of public and private healthcare providers.

Both countries have well-established service networks for people with SUDs, including harm reduction programs and specialized outpatient and inpatient addiction treatment services. OAT is offered in the Czech Republic and Norway with different levels of availability and affordability. In the Czech Republic, methadone is available in specialized OAT clinics with varying levels of threshold and is fully covered by public funds, while buprenorphine products can be prescribed by primary care physicians but require patients to pay full price. In Norway, OAT is considered low-threshold and harm reduction-oriented, and the costs are covered by the government.

### Data sources

This study utilized multiple population-based health registers from the Czech Republic and Norway. In both countries, physicians and other authorized healthcare professionals (public and private) are legally obliged to routinely report patient data to these nationwide registers. Information collected in the registers includes patient age and sex, admission and discharge dates, and the primary and secondary diagnoses. Diagnoses are recorded according to the International Classification of Diseases, 10th revision (ICD-10). In each country, data were linked across health registers using the unique personal identification numbers assigned to all people living in respective countries.

In the Czech Republic, the National Health Registers are curated by the publicly funded Institute of Health Information and Statistics. The *National Register of Addiction Treatment* (NRLUD) was used to identify OAT patients. It is a population-based health register covering demographic and treatment-related data of all patients entering the addiction treatment facilities in the Czech Republic. The NRLUD includes information on substance use disorder diagnosis, the treatment initiation date, and prescribed OAT medication. Information on physical diseases and other health conditions was obtained from the *National Register of Hospitalized Patients* (NRHOSP) and the *National Register of Reimbursed Health Service*s (NRHZS), which include hospitalizations and outpatient secondary (specialized) healthcare visits.

For Norway, *the Norwegian Prescription Database* (NorPD) provided data on OAT medications dispensed, dispensation date and amount measured as Defined Daily Doses [26]. Since Norway does not have a specific OAT registry, NorPD provided a proxy indication by identifying OAT patients based on filled prescriptions. This database identifies approximately 90% of OAT patients in Norway [27]. Opioids used for identification of OAT patients included methadone oral solution (Anatomical Therapeutic Chemical (ATC) code N07BC02) and high-dose buprenorphine tablets (≥ 2 mg sublingual tablets, N07BC01 (buprenorphine) or N07BC51 (buprenorphine-naloxone), all almost solely prescribed for the treatment of OUD. Physical morbidity data was obtained using *The Norwegian Patient Registry* (NPR) which contains information on hospitalizations and outpatient secondary (specialized) healthcare visits [28].

### Study population and study period

The study population included all individuals with at least one record of receiving OAT treatment in the Czech Republic and Norway between January 1, 2010–December 31, 2019. A total of 4,280 OAT patients from the Czech Republic and 11,389 OAT patients from Norway were included in the analysis. The mean age of patients was calculated based on their age at the midpoint of the follow-up period i.e., in 2015.

### Outcomes

Physical morbidity refers to the burden of diseases and conditions of all major organ systems and injuries. Injuries causing physical harm or damage to body tissues were included for their potential to result in long-term physical health consequences. Physical diseases and conditions were identified based on three-character diagnosis codes and categorized according to the ICD-10. In the analysis, we included all diagnoses that patients received during individual inpatient or outpatient treatment episodes that occurred between January 1, 2010–December 31, 2019, regardless of the date of the first OAT entry.

The following diagnosis chapters and their respective sections and codes were studied: A00-B99 Certain infections and parasitic diseases; C00-D48 Neoplasms; D50-D89 Diseases of blood and blood-forming organs and certain disorders involving the immune mechanism; E00-E90 Endocrine, nutritional and metabolic diseases; G00-G99 Diseases of the nervous system; H00-H59 Diseases of the eye and adnexa; H60-H95 Diseases of the ear and mastoid process; I00-I99 Diseases of the circulatory system; J00-J99 Diseases of the respiratory system; K00-K93 Diseases of the digestive system; L00-L99 Diseases of the skin and subcutaneous tissue; M00-M99 Diseases of the musculoskeletal system and connective tissue; N00-N99 Diseases of the genitourinary system; S00-T98 Injury, poisoning and certain other consequences of external causes. Overdose (T36-T50; ICD-10) was not singled out as a separate group as this was not the primary objective of this study.

### Statistical analysis

Descriptive statistics were used to describe and compare physical morbidity in the study population. Prevalence (%) of physical diseases was calculated as the number of OAT patients with at least one recorded diagnosis from respective chapters and sections divided by the total number of patients receiving OAT in the study period. Physical morbidity was described according to diagnosis chapters and sections and stratified by gender. Pearson’s chi-square test was used to test for differences in gender groups. Statistical analyses were performed by IBM SPSS Statistics 23.

### Ethics

This study was approved by the Ethics committees in the Czech Republic (no. 36/19GrantAZVVES20201.LFUK) and Norway (no. 2019/656/REC South-East C).

## Results

### Cohort characteristics

In total, 15,669 patients that received OAT in the Czech Republic (N = 4,280) and Norway (N = 11,389) between 2010 and 2019 was identified in the national registers. Table 1 shows the characteristics of the cohort. Both study cohorts had a majority of men (69.9% in the Czech Republic and 70.3% in Norway). The mean age was higher for both women and men in the Norwegian cohort (the Czech Republic: 35.8 for men and 33.0 for women; Norway: 42.8 for men and 41.8 for women). In the case of OAT medication, methadone prescription was similar in both countries, but the Czech cohort had higher buprenorphine prescribing and lower buprenorphine-naloxone prescribing compared to the Norwegian cohort.

**Table 1:** Characteristics of study population of opioid agonist treatment (OAT) patients in the Czech Republic and Norway during 2010–2019

	Czech Republic (N = 4,280)		Norway (N = 11,389)	
	Men	Women	Men	Women
Number of OAT patients (n, %)	2992 (69.9)	1288 (30.1)	8006 (70.3)	3383 (29.7)
Age at 2015				
Mean (SD)	35.8 (6.6)	33 (6.5)	42.8 (10.2)	41.8 (10.4)
First OAT medication				
Methadone (n, %)	946 (31.6)	446 (34.6)	2557 (31.9)	1142 (33.8)
Buprenorphine (n, %)	1225 (40.9)	512 (39.8)	2723 (34.0)	1215 (35.9)
Buprenorphine-naloxone (n, %)	821 (27.4)	330 (25.6)	2726 (34.0)	1036 (30.3)

SD = standard deviation

### Gender differences

**Fig. 1:**
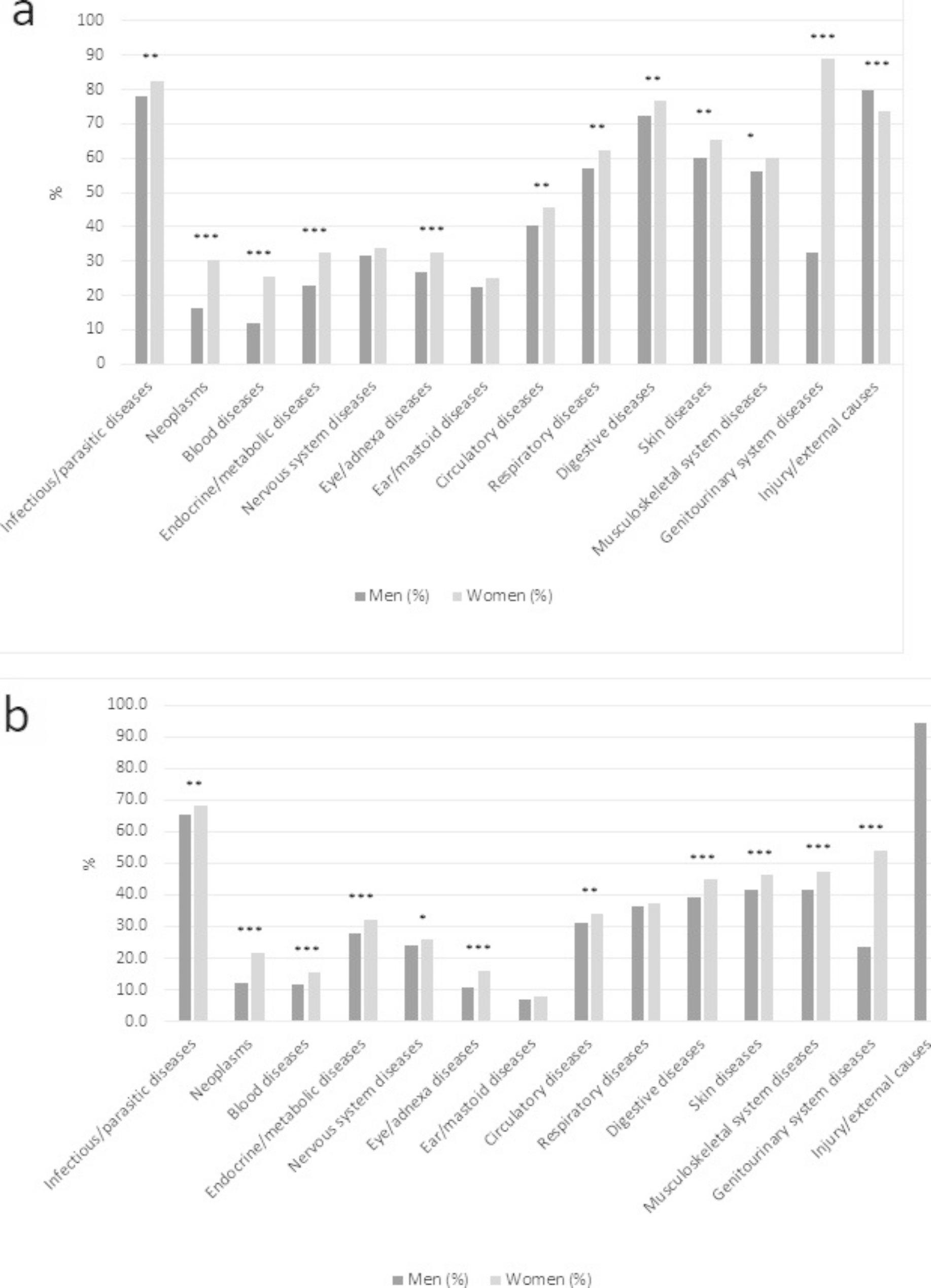
(**a**) Somatic diseases stratified by gender in opioid maintenance treatment patients in the Czech Republic during 2010–2019. (**b**) Somatic diseases stratified by gender in opioid maintenance treatment patients in Norway during 2010–2019 *p < .05 ** p < .01 ***p < .001

Overall, in both cohorts, OAT women had a significantly higher prevalence of physical diseases across most diagnostic chapters (Table 2; Fig. 1a and b). In the Czech cohort, an exception was injuries/external causes, which was significantly more prevalent in men (80.0% vs. 73.8%). Genitourinary diseases and neoplasms were approximately twice as high in women as in men in the Czech Republic and Norway. In addition, women in the Czech Republic showed over twice the prevalence of blood diseases (25.5% vs. 11.9%). Injuries/external causes and infectious/parasitic were the top two diseases in Czech men and Norwegian women and men. The prevalence of genitourinary diseases was high among women, especially in the Czech Republic (88.9% in the Czech Republic and 53.9% in Norway).

**Table 2:** Total prevalence of somatic diseases in opioid maintenance treatment patients in the Czech Republic and Norway during 2010–2019 by gender

		Czech Republic (N = 4,280)					Norway (N = 11,389)				
		Men (n = 2,992)		Women (n = 1,288)			Men (n = 8,006)		Women (n = 3,383)		
Chapter	ICD-10	n	%	n	%	p-value	n	%	n	%	p-value
Infectious/parasitic diseases	A00-B99	2339	78.2	1061	82.4	0.002	5236	65.4	2300	68.0	0.008
Neoplasms	C00-D48	490	16.4	391	30.4	< 0.001	958	12.0	739	21.8	< 0.001
Blood diseases	D50-D89	355	11.9	329	25.5	< 0.001	934	11.7	529	15.6	< 0.001
Endocrine/metabolic diseases	E00-E90	679	22.7	417	32.4	< 0.001	2222	27.8	1085	32.1	< 0.001
Nervous system diseases	G00-G99	945	31.6	435	33.8	0.164	1917	23.9	869	25.7	0.048
Eye/adnexa diseases	H00-H59	806	26.9	421	32.7	< 0.001	877	11.0	542	16.0	< 0.001
Ear/mastoid diseases	H60-H95	676	22.6	323	25.1	0.083	555	6.9	261	7.7	0.139
Circulatory diseases	I00-I99	1215	40.6	587	45.6	0.003	2492	31.1	1152	34.1	0.002
Respiratory diseases	J00-J99	1705	57.0	802	62.3	0.001	2928	36.6	1258	37.2	0.535
Digestive diseases	K00-K93	2170	72.5	987	76.6	0.006	3134	39.1	1514	44.8	< 0.001
Skin diseases	L00-L99	1795	60.0	840	65.2	0.001	3348	41.8	1566	46.3	< 0.001
Musculoskeletal system diseases	M00-M99	1682	56.2	775	60.2	0.017	3331	41.6	1607	47.5	< 0.001
Genitourinary system diseases	N00-N99	965	32.3	1145	88.9	< 0.001	1877	23.4	1825	53.9	< 0.001
Injury/external causes	S00-T98	2393	80.0	951	73.8	< 0.001	7538	94.2	3180	94.0	0.748

There was variation between countries. Injuries were the only condition where Norwegian women and men showed a higher proportion than Czech men (94.2% vs. 80.0%) and women (94.0% vs. 73.8%). In addition, Norwegian men had a slightly higher proportion of endocrine/metabolic diseases (27.8% vs. 22.7%). For men, the largest percentage-point differences between cohorts were in digestive diseases (33.4% points higher among men in the Czech Republic). For women, the largest percentage-point differences between cohorts were in genitourinary diseases (35.0% points higher among women in the Czech Republic).

The top three leading diagnosis groups for the five most prevalent chapters according to gender for the Czech Republic and Norway are shown in Supplementary Tables 1 and 2, respectively.

## Discussion

In the Czech Republic and Norway, women in OAT had a significantly higher prevalence of physical diseases across most diagnostic chapters, notably genitourinary diseases and neoplasms, compared to men. Injuries/external causes and infectious/parasitic diseases were among the most common diseases in both women and men.

Our finding that women in OAT are more burdened with physical diseases is consistent with previous studies reporting overall poorer health status [17, 24], higher rates of chronic diseases [9, 17], and higher hospitalization rates [24] in women receiving OAT. This could indicate that illicit opioid use is more harmful to women than men in terms of physical health, possibly due to a combination of multiple biological and socio-environmental factors [19, 22]. One of the reasons may be sex-based differences in the physiological response to illicit and pharmacological opioids. Women have higher blood drug concentrations and get intoxicated faster after equivalent doses due to different body composition (less body water, less muscle mass, and more fat mass), which contributes to the more harmful effects of opioids in women [29]. In addition, for the same reason, elevated and long-term exposure to other negative risk factors, such as alcohol use [30] and tobacco smoking [31, 32], may also contribute to a higher physical disease burden in OAT women [19, 22]. Out of socio-environmental factors, it has been shown that women are more likely to engage in economically motivated high-risk sexual behavior and needle sharing [33, 34]. This could explain the higher prevalence of infectious/parasitic diseases in particular. Therefore, it is important to consider multiple factors when addressing the impact of opioid use on the health of women in OAT.

In women from both cohorts, the prevalence of genitourinary diseases was roughly 2.5 times higher than in men. This dramatic difference may reflect a higher susceptibility of women to certain genitourinary conditions. One known risk of long-term opioid use is the development of endocrinopathy. By disrupting endocrine functions and dysregulation of sex steroid hormones, opioids can alter a menstrual cycle, which may lead to irregular periods or amenorrhea [35, 36]. Another reason could be the high prevalence of genitourinary tract infections in this population. These infections are among the most common bacterial infections in the general population and are significantly more prevalent in women due to women anatomy [37].

Gender-based disparities in healthcare-seeking behavior may account for some of the variations observed in disease prevalence rates. It is well-established that women generally tend to utilize primary and specialized healthcare services more frequently than men [38], which could potentially lead to higher diagnosis rates in women relative to men. On the contrary, higher morbidity rates may reflect greater barriers women face in accessing specialized addiction treatment, such as higher levels of perceived stigmatization and higher rates of co-morbid mental health problems [19, 39], which calls for higher support of women with OUD.

In both cohorts, infectious/parasitic were among the top three most common physical diseases in women and men, with viral hepatitis being the leading cause of burden that accounted for more than half of infectious morbidity. Infectious diseases, including HIV/AIDS, viral hepatitis, infectious endocarditis, and pneumonia, are the major cause of morbidity among people with opioid dependency [40, 41]. Of the infectious diseases classified in other ICD-10 diagnostic chapters, skin and soft tissue infections and respiratory infections were also common in the two cohorts. Skin and soft tissue infections are the common reason for hospital admissions in people injecting drugs as well as patients receiving injection OAT [42–44]. In addition to socio-environmental factors such as high-risk sexual behavior, poor nutritional status, poor hygiene, or drug and injection paraphernalia contamination, epidemiological studies suggest that opioid-induced immunosuppression could contribute to increased susceptibility to infections in people with OUD [45–47]. Moreover, women with OUD are at increased risk of infectious diseases due to sex work, with many continuing to be engaged even after entering OAT [48]. In general, to prevent and decrease the rates of infectious diseases in this population, OAT and low-threshold services are considered essential [49]. Supporting OAT retention and the harm reduction approach should be therefore a priority in addressing infectious diseases among people with OUD.

Injuries/external causes were the top leading condition in Norwegian women and men and Czech men. The high prevalence of injuries/external causes is likely due to drug-induced alteration in psychomotor functioning, resulting in impaired coordination and judgment, increased risk-taking and violent behavior, and self-harm [50]. Irrespective of the cause, injuries and external causes are preventable. Thus, a great amount of effort should be invested in the reduction of risks associated with these problems.

Although cancer incidence is generally higher in men in Europe [51], neoplasms were almost twice as common in OAT women as in men in this study. This higher prevalence of neoplasms among women supports an unequal distribution in exposure to risk factors associated with cancer in favor of women. In addition to the biological and environmental risk factors discussed above, the higher incidence of women-specific cancers, such as female breast, ovarian, and cervical cancers, may have contributed to higher rates of neoplasms in OAT women. However, the evidence on gender differences and cancer risk in opioid-dependent people is scarce so far. Some recent studies investigating people with prolonged use of opium suggest an opposite tendency, which is a higher prevalence of neoplasms in men [52, 53]. Future research investigating women-specific neoplasms could help explain the higher prevalence of neoplasms in women with OUD.

This study did not compare the morbidity of OAT patients with the general non-dependent population. However, a similar study found significantly increased rates of mortality, hospital admissions, and ED visits in Australian patients with OUD even after entering OAT. Morbidity was significantly elevated in patients receiving OAT, particularly women, even for non-drug-related causes i.e., infectious, cardiovascular, blood, endocrine/metabolic, digestive, nervous, genitourinary, respiratory, and musculoskeletal diseases [6]. Although, based on the previous findings, we could anticipate higher disease prevalence rates in our OAT population compared to the general Czech and Norwegian populations, additional evidence is necessary to validate this assumption.

Globally, cardiovascular diseases are the leading cause of disease burden among the general population [54]. In this study, circulatory disease prevalence was lower compared to other diagnostic chapters, but still relatively high. Although the biological pathways are not yet fully understood, several studies associated opioid use with an increased risk of cardiovascular diseases [55, 56]. Infective endocarditis and thrombosis are known cardiovascular complications associated with injection drug use [57]. Moreover, methadone exposure has been linked with an increased risk of cardiotoxicity, specifically QT prolongation and torsade de pointes [58, 59]. A Danish registry-based study found intravenous drug use and methadone use to be associated with a higher incidence of cardiovascular diseases [55].

Increased exposure to illicit opioids, as well as other illicit drugs and health risk factors (e.g., alcohol use, smoking, poor diet) from a very young age in individuals with OUD have been shown to trigger the early onset of diseases that usually manifest in later life due to cumulative substance-related organ toxicity and untreated complex health conditions [60–62]. When investigating physical morbidity in the opioid-use population, we should consider that even diseases and conditions usually related to higher age may cause a physical burden in young OAT patients. Therefore, special attention should be paid to the early detection and treatment of physical diseases in order to prevent disease progression and improve the health outcomes of the ageing OAT population.

Finally, it is worth noticing that this study only covered secondary healthcare and hospitalization records. We might therefore expect diseases that added to the prevalence estimates to be of greater severity than conditions usually treated by primary care physicians. On the other hand, the prevalence of physical diseases is likely to be underestimated for the same reason.

### Strengths and limitations

The strength of this study is the gender-specific nationwide registry-based design allowing for a large and unselected study sample with high statistical power. Utilizing data from two countries with compatible health registers and similarities in healthcare and OAT systems increases the generalizability of findings. Recall bias was minimized by using prospectively collected data [63].

Some known limitations of registry-based studies that could affect this study validity include missing data and unknown data accuracy and quality due to the variation in coding practices between different individuals reporting to the registers [63]. This study did not consider the patterns and duration of opioid use, the severity of OUD, and the duration of OAT in patients. It is therefore possible, that to some extent, the variation in physical morbidity may be attributed to differences in substance use practices between women and men. For example, women may take higher doses of opioids for a longer time, use riskier route of drug administration, use other substances, or receive OAT for a shorter period than men. The large differences between the two countries in the prevalence of physical diseases may be partially attributed to the different use of data sources in each country. While the patient data stored in the registers are similar, the registers are created, maintained, and administered by each country independently. For data protection and legal reasons, merging the registry datasets of the Czech Republic and Norway was not feasible in this study. In addition, since Norway does not have a specific OAT registry, the Norwegian cohort could only be identified based on prescriptions by proxy indication from NorPD. In the Czech Republic, OAT patients have been selected directly from the OAT registry – the NRLUD. In addition, the age-stratified analysis was not performed in this study. Given that the burden of physical diseases increases with age, the total prevalence of diseases may vary for different age groups.

## Conclusions

This study found a large burden of physical morbidity across gender groups in OAT patients. In particular, special medical attention should be given to women in OAT as they appear to be more vulnerable to physical diseases than men.

Intense and prolonged exposure to multiple negative risk factors likely contributes to high physical morbidity in OAT patients. Given that individuals with OUD are aging, an increase in physical morbidity, including chronic diseases, can be expected in this population.

Our findings support the need for early screening, detection, and evidence-based treatment of diseases and conditions across organ systems. More preventive efforts are needed to reduce the additional morbidity burden. Integration of health promotion activities such as alcohol reduction, smoking cessation, healthy diet, and physical activity is necessary to reduce future physical morbidity and extend the longevity and quality of life of OAT patients. The gender differences underline the need for a gender-specific and tailored approach to treating medical conditions to improve health outcomes in OAT patients.

## Electronic supplementary material

Below is the link to the electronic supplementary material.

Supplementary Material 1

## Data Availability

The authors are not authorized to share data analyzed in this study externally without a permission of the data provider in each country.

## Abbreviations

ATC: Anatomical Therapeutic Chemical
ED: Emergency Department
ICD-10: International Classification of Diseases, 10th Revision
NorPD: Norwegian Prescription Database
NPR: Norwegian Patient Registry
NRHOSP: National Register of Hospitalized Patients
NRHZS: National Register of Reimbursed Health Services
NRLUD: National Register of Therapy of Drug Users
OAT: Opioid Agonist Treatment
OUD: Opioid Use Disorder
SUD: Substance Use Disorder
