# Supplementary material for: Gender differences in physical morbidity in opioid agonist treatment patients: population-based cohort studies from the Czech Republic and Norway
Source: Subst Abuse Treat Prev Policy. 2023 Jul 28;18:47. doi: 10.1186/s13011-023-00557-8 (PMC10385939; doi:10.1186/s13011-023-00557-8)
Supplement: Supplementary file 1 — Supplementary Material 1 [file 13011_2023_557_MOESM1_ESM.docx]

**Supplementary Table 1** Top three leading diagnosis sections for the five most prevalent diagnostic chapters (ICD-10) in opioid maintenance treatment patients in the Czech Republic during 2010–2019 by gender

|  | **Men (n = 2,992)** | **Women (n = 1,288)** | | | | | |
| --- | --- | --- | --- | --- | --- | --- | --- |
| **Chapter** | **Section** | **n** | **%** | **Section** | **n** | **%** |  |
| Infectious/parasitic diseases (A00-B99) |  |  |  |  |  |  |  |
|  | Viral hepatitis (B15-B19) | 2025 | 67.7 | Viral hepatitis (B15-B19) | 889 | 69.8 |  |
|  | Other bacterial diseases (A30-A49) | 400 | 13.4 | Other bacterial diseases (A30-A49) | 192 | 14.9 |  |
|  | Mycoses (B35-B49) | 250 | 8.4 | Infections with a predominantly sexual mode of transmission (A50-A64) | 180 | 14.0 |  |
| Respiratory diseases (J00-J99) |  |  |  |  |  |  |  |
|  | Acute upper respiratory infections (J00-J06) | 1064 | 35.6 | Acute upper respiratory infections (J00-J06) | 564 | 43.8 |  |
|  | Influenza and pneumonia (J09-J18) | 559 | 18.7 | Influenza and pneumonia (J09-J18) | 248 | 19.3 |  |
|  | Other acute lower respiratory infections (J20-J22) | 390 | 13.0 | Other acute lower respiratory infections (J20-J22) | 226 | 17.5 |  |
| Digestive diseases (K00-K93) |  |  |  |  |  |  |  |
|  | Diseases of esophagus, stomach and duodenum (K20-K31) | 1178 | 39.4 | Diseases of esophagus, stomach and duodenum (K20-K31) | 567 | 44.0 |  |
|  | Diseases of oral cavity, salivary glands and jaws (K00-K14) | 1045 | 34.9 | Diseases of oral cavity, salivary glands and jaws (K00-K14) | 522 | 40.5 |  |
|  | Diseases of liver (K70-K77) | 916 | 30.6 | Diseases of liver (K70-K77) | 366 | 28.4 |  |
| Skin diseases (L00-L99) |  |  |  |  |  |  |  |
|  | Infections of the skin and subcutaneous tissue (L00-L08) | 1377 | 46.0 | Infections of the skin and subcutaneous tissue (L00-L08) | 616 | 47.8 |  |
|  | Dermatitis and eczema (L20-L30) | 602 | 20.1 | Dermatitis and eczema (L20-L30) | 342 | 26.6 |  |
|  | Other disorders of the skin and subcutaneous tissue (L80-L99) | 338 | 11.3 | Disorders of skin appendages (L60-L75) | 222 | 17.2 |  |
| Injury/external causes (S00-T98) |  |  |  |  |  |  |  |
|  | Injuries to the head (S00-S09) | 1276 | 42.6 | Injuries to the head (S00-S09) | 449 | 34.9 |  |
|  | Injuries to the wrist and hand (S60-S69) | 1223 | 40.9 | Injuries to the wrist and hand (S60-S69) | 396 | 30.7 |  |
|  | Injuries to the ankle and foot (S90-S99) | 882 | 29.5 | Injuries to the ankle and foot (S90-S99) | 323 | 25.1 |  |

**Supplementary Table 2** Top three leading diagnosis sections for the five most prevalent diagnostic chapters (ICD-10) in opioid maintenance treatment patients in Norway during 2010–2019 by gender

|  | **Men (N = 8,006)** | **Women (N = 3,383)** | | | | | | |
| --- | --- | --- | --- | --- | --- | --- | --- | --- |
| **Chapter** | **Section** | | **n** | **%** | **Section** | | **n** | **%** |
| Infectious/parasitic diseases (A00-B99) |  | |  |  |  | |  |  |
|  | Viral hepatitis (B15-B19) | | 4447 | 55.5 | Viral hepatitis (B15-B19) | | 1873 | 55.4 |
|  | Other bacterial diseases (A30-A49) | | 1892 | 23.6 | Other bacterial diseases (A30-A49) | | 781 | 23.1 |
|  | Intestinal infectious diseases (A00-A09) | | 319 | 4.0 | Intestinal infectious diseases (A00-A09) | 168 | | 5.0 |
| Digestive diseases (K00-K93) |  | |  |  |  | |  |  |
|  | Diseases of esophagus, stomach and duodenum (K20-K31) | | 1064 | 13.3 | Other diseases of intestines (K55-K64) | | 557 | 16.5 |
|  | Other diseases of intestines (K55-K64) | | 966 | 12.1 | Diseases of esophagus, stomach and duodenum (K20-K31) | | 437 | 12.9 |
|  | Diseases of liver (K70-K77) | | 582 | 7.3 | Disorders of gallbladder, biliary tract and pancreas (K80-K87) | | 291 | 8.6 |
| Skin diseases (L00-L99) |  | |  |  |  | |  |  |
|  | Infections of the skin and subcutaneous tissue (L00-L08) | | 2531 | 31.6 | Infections of the skin and subcutaneous tissue (L00-L08) | | 1042 | 30.8 |
|  | Other disorders of the skin and subcutaneous tissue (L80-L99) | | 830 | 10.4 | Other disorders of the skin and subcutaneous tissue (L80-L99) | | 507 | 15.0 |
|  | Dermatitis and eczema (L20-L30) | | 344 | 4.3 | Dermatitis and eczema (L20-L30) | | 221 | 6.5 |
| Musculoskeletal system diseases (M00-M99) |  | |  |  |  | |  |  |
|  | Other soft tissue disorders (M70-M79) | | 1690 | 21.1 | Other soft tissue disorders (M70-M79) | | 843 | 24.9 |
|  | Other dorsopathies (M50-M54) | | 849 | 10.6 | Other joint disorders (M20-M25) | | 488 | 14.4 |
|  | Other joint disorders (M20-M25) | | 819 | 10.2 | Other dorsopathies (M50-M54) | | 428 | 12.7 |
| Injury/external causes (S00-T98) |  | |  |  |  | |  |  |
|  | Injuries to the head (S00-S09) | | 2396 | 29.9 | Injuries to the head (S00-S09) | | 730 | 21.6 |
|  | Injuries to the wrist and hand (S60-S69) | | 1955 | 24.4 | Injuries to the wrist and hand (S60-S69) | | 598 | 17.7 |
|  | Poisoning by, adverse effect of and under-dosing of drugs, medicaments and biological substances (T36-T50) | | 1641 | 20.5 | Poisoning by, adverse effect of and under-dosing of drugs, medicaments and biological substances (T36-T50) | | 659 | 19.5 |
